# Anticancer activity of *Caesalpinia sappan* by downregulating mitochondrial genes in A549 lung cancer cell line

**DOI:** 10.12688/f1000research.76187.1

**Published:** 2022-02-11

**Authors:** Nashi Widodo, Sapti Puspitarini, Muhammad Hermawan Widyananda, Adzral Alamsyah, Septian Tri Wicaksono, Masruri Masruri, Yoga Dwi Jatmiko

**Affiliations:** 1 Biology Department, Faculty of Mathematics and Natural Sciences, Brawijaya University, Malang, Indonesia; 2 Faculty of Mathematics and Natural Sciences, Brawijaya University, Malang, Indonesia; 3 Chemistry Department, Faculty of Mathematics and Natural Sciences, Brawijaya University, Malang, Indonesia

**Keywords:** A549 cells, apoptosis, ATP production, Caesalpinia sappan, cytotoxicity, mitochondrial dysfunction

## Abstract

**Background:** The standardization and mechanism of action of *Caesalpinia sappan* as an anticancer agent are still lacking. This study aimed to understand the mechanism of action of *C,sappan* extract as an anticancer agent.

**Methods:** This study was conducted using the A549 lung cancer cell line to understand the mechanism of action of *C. sappan* extract as an anticancer agent. The cytotoxicity activity, cell cycle progression, apoptosis, protein-related apoptosis (i.e., BCL-2and BAX protein) assays, and RNA sequencing were performed level were measured. Moreover, the antioxidant activity, total flavonoids, and phenolics of *C.sappan* were also assessed.

**Results:** *C.sappan* has strong antioxidant activity (22.14 ± 0.93 ppm) total flavonoid content of (529.3 ± 4.56 mgQE/g), and phenolics content of (923.37 ± 5 mgGAE/g). The *C.sappan* ethanol extract inhibited cancer cell growth and arrested at G0/G1 phase of cell cycle, inducing apoptosis by increasing BAX/BCL-2 protein ratio in A549 lung cancer cell line. Furthermore, results from RNA sequencing analysis showed that *C.sappan* ethanol extract caused downregulation of genes acting on mitochondrial function including adenosine triphosphate (ATP) production and respiration.

**Conclusions:** This study demonstrated that *C.sappan* has the ability to inhibit cancer cell growth by inducing apoptosis and mitochondrial dysfunction in A549 cells.

## Introduction

Lung cancer is one of the most dangerous and leading cancer killers globally. ^1^ In 2020, there were 247,270 new cases of lung cancer in the United States, wherein 130,430 and 116,930 cases were from man and women, respectively. Additionally, there were 140,7230 deaths from lung cancer, in which 76,370 and 64,360 were man and women, respectively. ^2^ Lung cancer is the 14 ^th^ leading cause of death in Indonesia. Based on the WHO data (2018), the death rate due to lung cancer in Indonesia is 33,296, and there were 29,921 deaths from March to November 2020. Lung cancer is the 14 ^th^ leading cause of death in Indonesia. ^3^

Lung cancer treatment still relies on radiation and chemotherapy, especially for the treatment of metastatic lung cancer. ^4^^,^^5^ Current treatment regiments for lung cancer includes drugs such as gefitinib, nivolumab, and erlotinib or monoclonal antibodies (cetuximab, bevacizumab), which block the growth and spread of cancer by interfering with specific molecules involved in tumor growth and development. ^6^ However, these treatments are relatively expensive; hence, using traditional herbal medicine is an alternative option. The development of herbal medicines as anticancer agents not yet been explores, and it lacks standardization. Thus, it is essential  to study the mechanism of action of herbs that have been empirically used as cancer treatments for the development of phytopharmaceuticals. ^7^

Secang ( *Caesalpinia sappan*), a plant from the Caesalpiniaceae family which is often used as a traditional drink and natural dye in certain regions of Indonesia, primarly using the heartwood. Secang is known to contain the active compound brazilin in addition to various other compounds that contain antitumor, antibacterial, antiviral, antioxidant, and immunostimulant properties. ^5^^,^^8^ However, standardization and development of the use of *C. sappan* as an anticancer agent are still lacking. Therefore, this study aimed to analyze the effect of ethanol extract of secang ( *C. sappan*) on lung cancer cell culture (A549 cell line) in inhibiting cell growth and study its mechanism.

## Methods

### Plant and extraction

Dry powder of *C. sappan* stem was purchased from UPT Materia Medica Batu, Batu, Indonesia. The *microwave-assisted extraction* method was used for powder extraction by dissolving the powder in ethanol in a 1:10 ratio (herb: solvent; w/v) using a holding temperature of 50°C, warming at 50°C for 5 mins, holding time of 10 mins, cool down of 5 mins, and power of 1500 W. The extract was filtered using filter paper and then evaporated using a rotary vacuum evaporator (Buchi: Buchi R-210 Rotavapor System). The extract was freeze-dried until a dried extract was obtained.

### Cytotoxicity assay

The cytotoxicity activity of *C. sappan* ethanol extract was tested using WST-1 (Sigma: 11644807001) with 5% concentration (v/v medium). The A549 cells were obtained from the Japanese Collection of Research Bioresources Cell Bank, Japan. The A549 cells were seeded on 96-well plates to approximately 7.500 cells/well. The concentrations of *C. sappan* ethanol extract used were 10 μg/mL, 20 μg/mL, 40 μg/mL, 80 μg/mL, 160 μg/mL, and 320 μg/mL. Then the cells were treated for 24 h. The absorbance was measured using an ELISA reader (BioTek ELx808) at a wavelength of 450 nm. Cell viability was adjusted using Equation (1). The IC _50_ value was calculated from linear regression of the cytotoxicity chart using Equation (2).

$$\%\text{viability of cells}:\left(\frac{\text{Abstreatment}-\text{Absblanko}}{\text{Abscontrol}-\text{Absblanko}}\right)\times 100\%$$ (1)

$$Y=\mathrm{ax}+b,\text{where}\;Y=50\%;\;x={\mathrm{IC}}_{50}\;\text{dose}$$ (2)

### Cell cycle, apoptosis, and BAX/BCL-2 ratio assay

The cells were seeded to approximately 75.000 cells/well into 24-well plates. The doses used in the cell apoptosis assay were obtained from the IC _50_ value. After incubation for 24 h, the treated A549 cells were harvested and incubated with the dyes for 30 min. The cells were then analyzed using the flow cytometer (BD FACSCalibur) with CellQuest software version 6.0. The cells were dyed using propidium iodide (BioLegend: 421301) for cell cycle assay and tested using annexin-V/propidium iodide dyes (BioLegend: 640932) for the cell apoptosis assay. For the BAX/BCL-2 assay, the cells were tested using BAX (SantaCruz: sc-20067) and BCL-2 (SantaCruz: sc-20067) antibodies.

### 2,2–Diphenyl-1-picrylhydrazyl (DPPH) scavenging activity assay

Approximately 100 μL of *C. sappan* ethanol extract solution was added to 100 μL DPPH (Sigma: D9132) solution with 0.4 mM concentration in 96-well plates. The sample was incubated for 30 min, and absorbance was measured using an ELISA reader (BioTek ELx808) with λ = 490 nm. The inhibition percentage of scavenging activity was adjusted using Equation (3). The IC _50_ value was calculated from linear regression of the cytotoxicity chart using Equation (2).

$$\%\text{inhibiton}:\left(\frac{\text{Abstreatment}-\text{Absblanko}}{\text{Abscontrol}-\text{Absblanko}}\right)\times 100\%$$ (3)

### Total flavonoids assay

Total flavonoids were estimated using the aluminum chloride colorimetric assay. ^9^ Quercetin (MarkHerb) was used as the standard. Approximately 50 μL of *Caesalpinia sappan* ethanol extract or standard was added to 10 μL of AlCl _3_ (Smartlab: A-2070) (10% w/v) followed by 150 μL of 96% ethanol solution (SmartLab). The solution was then added to 10 μL of CH _3_COONa (Sigma) with a 1M concentration. The solution was incubated at room temperature for 40 min and protected from light. The absorbance was measured using an ELISA reader (BioTek ELx808) at a wavelenght of λ = 405 nm. Total flavonoid contents were expressed in terms of quercetin equivalent (standard curve equation of quercetin; $y=0.0023+0.0583\;R^{2}=0.9992$ ) mg QE/g of dry extract.

### Total phenolic assay

The total phenolic content was estimated using the Folin–Ciocalteu. A sample or standard of 100 μL was added to 1.0 mL of the Folin–Ciocalteu reagent (Merck: 1090010500) (a ready-to-use reagent diluted tenfold with distilled water). After 5 min, 1.0 mL of Na _2_CO _3_ (Merck: 1063921000) (7.5% w/v) was added to the mixture and incubated at room temperature for 90 min in dark conditions. Total phenolic content was measured using spectrophotometry in λ = 725 nm. Gallic acid (MarkHerb) was used as standard. Gallic acid was expressed in terms of gallic acid equivalent (standard curve equation of gallic acid) mg GAE/g of dry extract.

### RNA extraction, preparation, and transcriptome sequencing

The cells were seeded with a density of 2.2 × 10 ^6^ cells in a 100-mm dish. The doses used in the cell apoptosis assay were obtained from the IC _50_ value and incubated for 24 h. RNA extraction processing was conducted at PT Genetika Science, Jakarta. Briefly, total RNA was extracted using the Quick RNA MiniPrep Kit (Zymo Research, R1057) as per the manufacturer’s instructions. The integrity, contaminant, quantitation, and purity of RNA samples were evaluated before further use. The preparation and transcriptome sequencing of RNA was conducted at NovogeneAIT Genomics, Singapore. Briefly, the library preparation for Eukaryotic mRNA using polyA enrichment method (NEB). Sequencing was created using Illumina NovaSeq 6000 PE150.

## Results

### Antioxidant activity, total phenol, and flavonoid content

Table 1 presents the antioxidant activity and total phenol and flavonoid content of *C. sappan.* ^39^ The results showed that *C. sappan* ethanol extract contains an abundant amount of phenol and flavonoid. Besides that, the antioxidant activity of *C. sappan* ethanol extract has low IC _50_ or strong activity of antioxidant. Bioactive compounds of *C. sappan* include phenol and flavonoid groups which have pharmacological activities, such as antioxidant and anticancer properties.

**Table 1.** Antioxidant activity and total phenol and flavonoid content of the samples. DPPH=2,2–Diphenyl-1-picrylhydrazyl.

Sample	IC _50_ of DPPH scavenging (ppm)	Total phenol (mgGAE/g)	Total flavonoid (mgQE/g)
*Caesalpinia sappan*	22.14 ± 0.93	923.37 ± 5	529.3 ± 4.56

### Anticancer activity of *C. sappan* against A549 cells line

**Figure 1.**
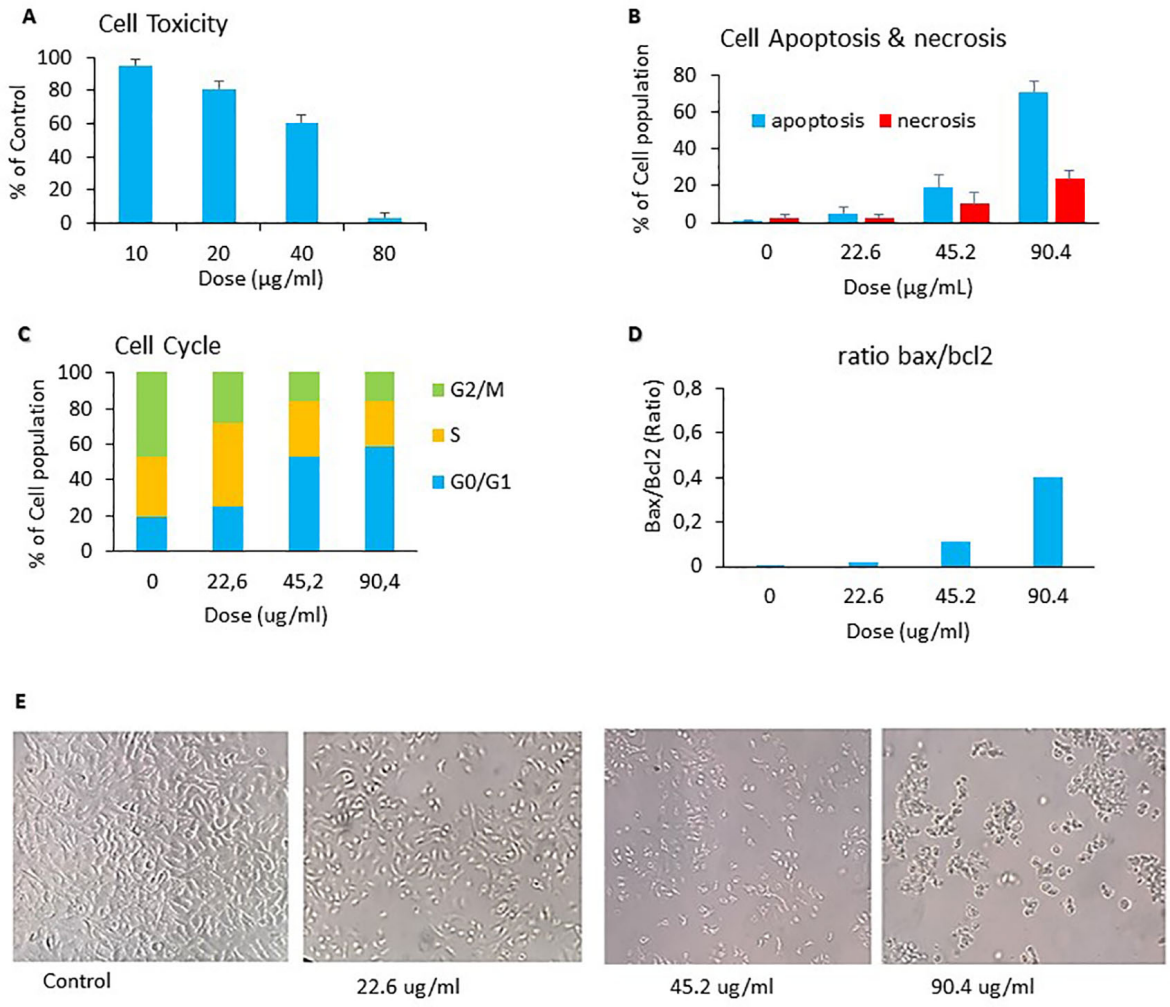
Anticancer activity of *C. sappan* against A549 cells line. (A) Cytotoxicity assay; (B) apoptosis assay; (C) cell cycle assay; (D) BAX-BCL-2 assay; (E) cell morphology microcopies.

The anticancer activity of *C. sappan* ethanol extract against A549 cells line was determined from cytotoxicity effect, capability inducing apoptosis, inhibiting cell cycle phase, and Bax/Bcl2 ratio. The cytotoxicity effect of *C. sappan* ethanol extract on A549 cells confirmed that *C. sappan* ethanol extract could inhibit the growth of A549 cells ( Figure 1A). The calculated IC _50_ value from Figure 1A was around 45.19 ± 1.704 μg/mL. The IC _50_ value was used as basic concentration for apoptosis, cell cycle, and Bax/Bcl2 assays. The extract’s capability to induce apoptosis on A549 cells was determined by annexin-V/PI dyes and analyzed by flow cytometry. The apoptosis assay showed that the treatment of *C. sappan* ethanol extract on A549 cells for 24 h indicated that the apoptotic cell percentage was increased compared with controls ( Figure 1B). The ability of *C. sappan* ethanol extract to inhibit the cell cycle in A549 cells was evaluated from cell cycle assay using PI dyes. The result indicated that the extract could induce cell cycle arrest in G0/G1 compared with control ( Figure 1C). The Bax/Bcl2 assay was conducted to analyze the protein associated with  to apoptosis. The level of  Bax increased compared with that of the control after 24 h of incubation with *C. sappan* ethanol extract. Moreover, theBcl2 level decreased compared with that of the control after 24 h of incubation with the extract. The result demonstrated that the *C. sappan* ethanol extract could induce apoptosis in A549 cells by increasing the Bax/Bcl2 protein ratio ( Figure 1D). Cell morphology observations showed results that were directly proportional to the anticancer activity assay. The population of cells in the control group (0 μg/mL) has a normal form of cancer cells. The 22.6 μg/mL group reduced the cell population density compared with the control group, whereas the 45.2 μg/mL group reduced the cell density and altered cell shape to smaller and rounder cells, indicating cell death. The 90.4 μg/mL group had dominant dead cells ( Figure 1E).

### Effect of *C. sappan* extract on the gene expression profile of A549 cells

**Figure 2.**
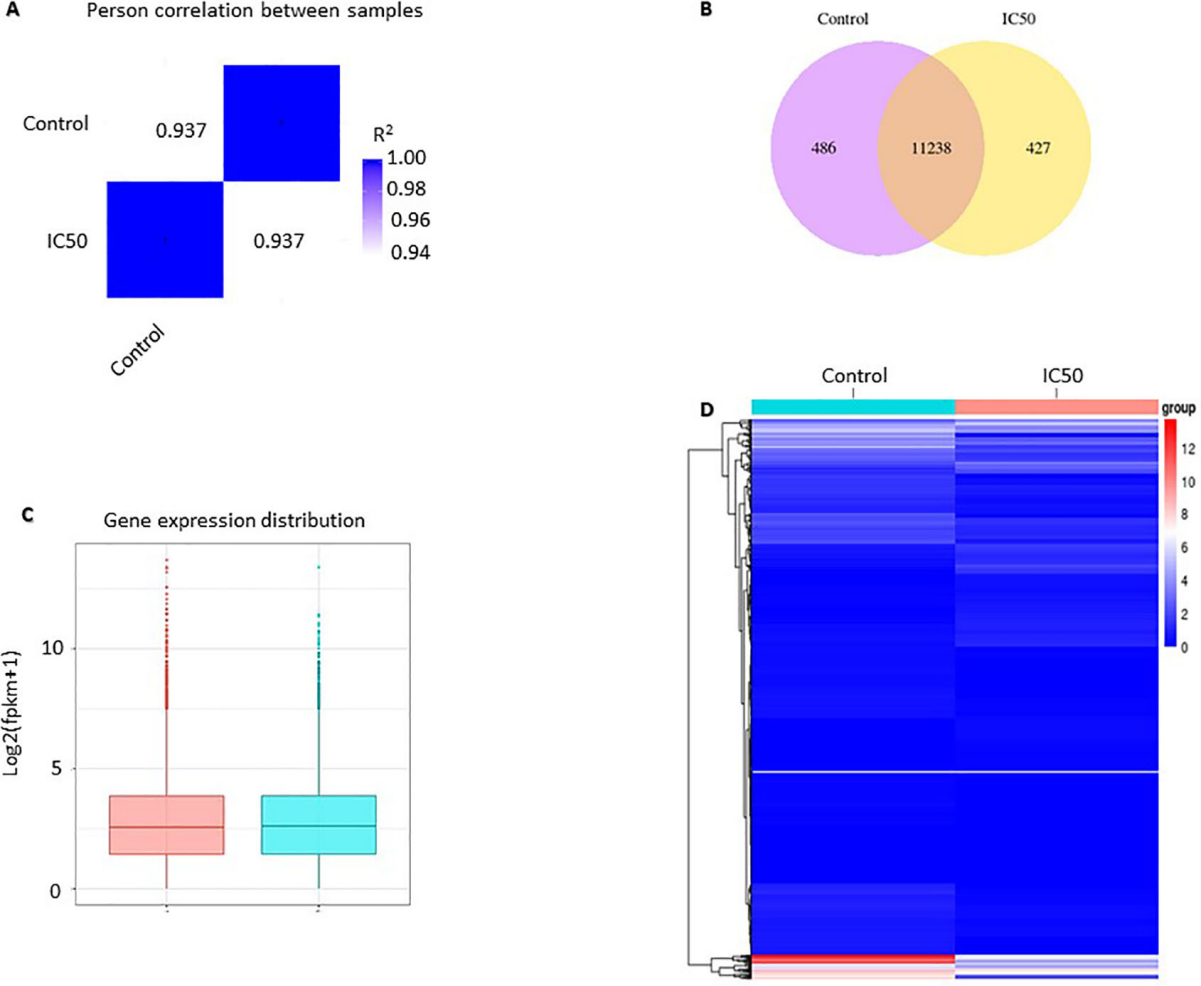
Gene expression profile of A549 cells after *C. sappan* extract treatment for 24 h. (A) Pearson correlation; (B) co-expression Venn diagrams; (C) gene expression distribution; (D) heatmap analysis.

RNA-seq analysis results showed differences in gene expression profiles between the control and those treated with the  extractat its IC _50_ value. Pearson correlation was used to verify the results of RNA sequencing, in which the replication was reliable and the sample selection was correct. Pearson correlation was constructed from all gene expression data (FPKM) in each sample. The R2 value of the same sample is 1, indicating a perfect correlation which means that the method and sample used are correct. The value of R2 between the control and IC _50_ is 0.937, which means that it is strongly correlated but not identical. The correlation value of 0.937 indicates a difference in gene expression between the control and IC _50_ ( Figure 2A). The analysis of co-expression Venn diagrams showed that 486 genes were expressed in the control group, 427 genes were expressed in *C. sappan* extract treatment, and 11238 genes were co-expressed in both groups ( Figure 2B). Box and whisker plot were constructed to compare the distribution of gene expression in the two samples. The results showed a not-too-distant difference overall. The difference is seen in the value of log2(fpkm+1) 10, indicating a difference in expression between samples ( Figure 2C). Comparison of gene expression between the control and IC _50_ can also be seen in the gene expression heatmap. The gene expression heatmap was constructed using Log2(FPKM+1) values summed with color; red and blue indicate high expression and low expression, respectively. From the heatmap, it can be observed that there are differences in gene expression between the control and IC _50_ ( Figure 2D).

### Changes in gene expression profile due to the extract treatment affect the biological processes of A549

**Figure 3.**
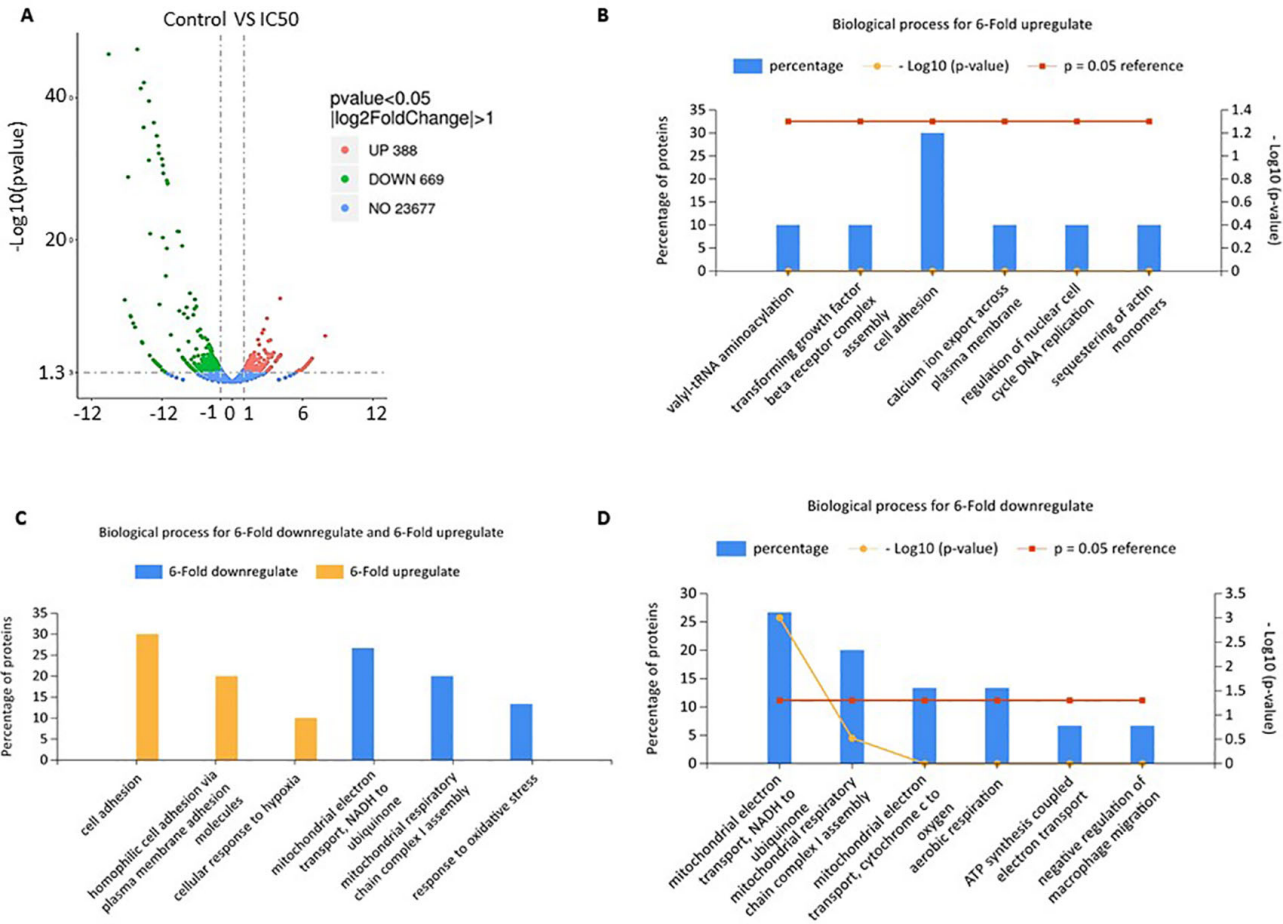
Gene expression profile affects the biological processes of A549 cells after *C. sappan* extract treatment for 24 h. (A) Volcano plot; (B) biological processes for sixfold upregulation; (C) biological processes for sixfold up- and downregulation; (D) biological processes for sixfold downregulation.

Volcano plot of gene expression from RNA-seq analysis showed that *C. sappan* extract treatment of A549 cells resulted in the upregulation of 388 genes and downregulation of 669 genes compared with the control group ( Figure 3A). Functional annotations were constructed from genes that had a sixfold increased expression and those that had a sixfold decreased expression compared with controls. The genes that have decreased expression have a role in adenosine 5'-triphosphate (ATP) synthesis and mitochondrial respiration ( Figure 3C and D). This indicates a decrease in the production of ATP by the mitochondria. This decrease in ATP production is thought to cause apoptosis and cell cycle arrest in A549 cells. Further, genes that experience an extreme increase in expression are involved in the process of hemostasis and attachment as the cells attempt to survive ( Figure 3B and C). The increase in some genes levels involved in the cell growth is the cell survival mechanism in response against the toxicity of the extract. However, these cell survival efforts were insufficient due to ATP depletion, so the cells experienced apoptosis, as evidenced by previous results.

### Network analysis of sixfold up- and downregulated genes

**Figure 4.**
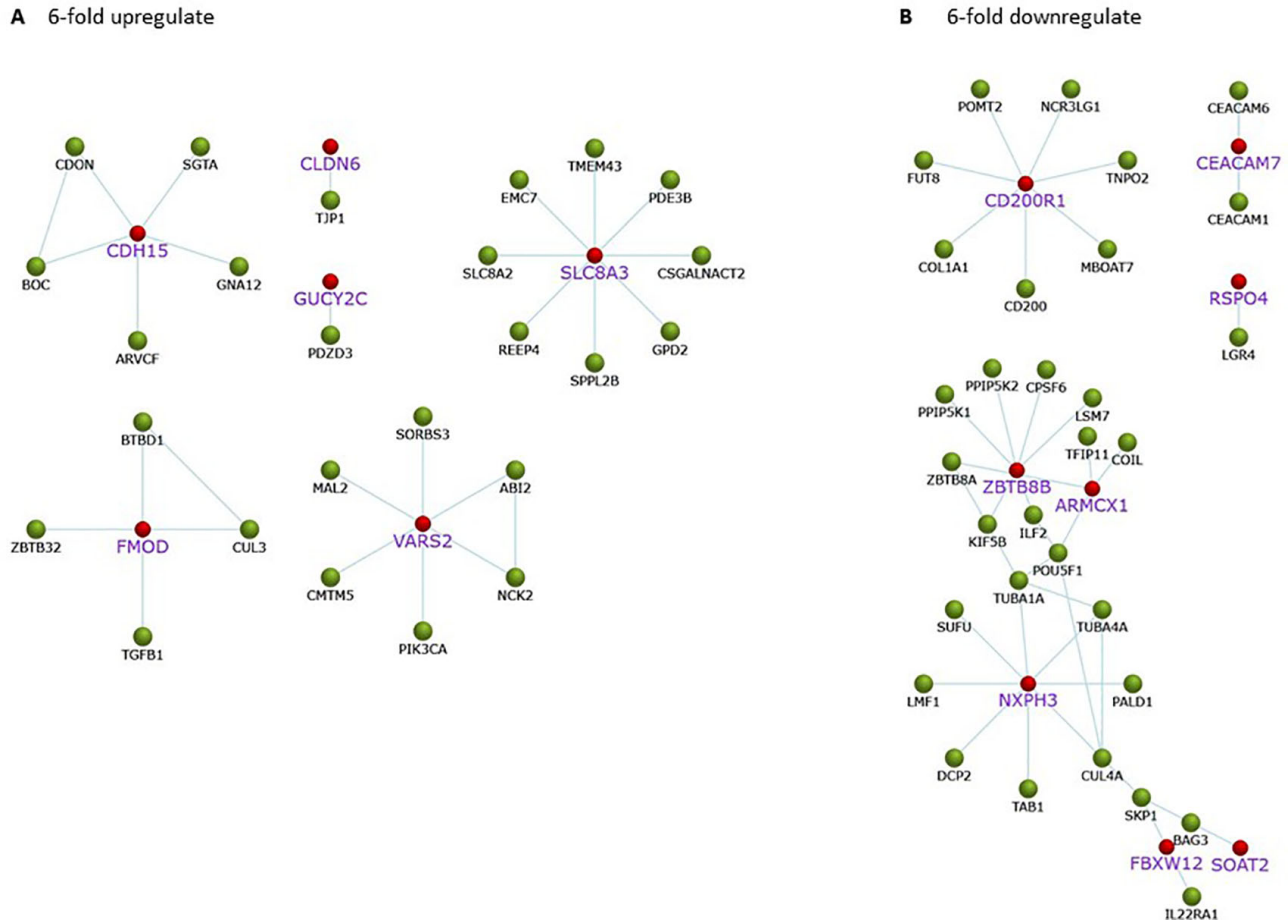
Protein interactions related to cancer on A549 cells after *C. sappan* extract treatment for 24 h. The red mark for gene found upregulated or downregulated in the A549 cells after treatment with the extract. (A) 6-fold upregulate; (B) 6-fold downregulate.

Gene expression of A549 cells after treatment with *C. sappan* extract for 24 h is correlated with protein interactions that play a role in biological processes associated with cancer. Upregulated proteins play a role in cell adhesion, namely, CDH15, CLDN6, and FMOD. In addition, there are also proteins, e.g., SLCA8A and GUCY2C, that interact with PDZD3, which play a role in maintaining intracellular ion homeostasis ( Figure 4A). Although the proteins associated with cell survival are upregulated, the cells will die if the ATP concentration drastically drops. Meanwhile, proteins that play a role in the regulation of mitochondrial work are downregulated, including ARMCX1. In addition to proteins related to ATP production, proteins whose interactions with other proteins affect the growth of cancer cells are downregulated, including CEACAMP7 and RSPO4 that play a role in cancer cells; ZBTB8B that involved in cancer cell development; NXPH3 interacts with proteins associated with microtubule formation and metastasis; SOAT2 and FBXW12 proteins that interact with the cancer cell cycle and growth-related proteins ( Figure 4B).

## Discussion

The ability of a substance as an anticancer agent can be seen from its ability to eliminate cancer cells. Cell death can be seen from various characteristics, including decreased cell viability, inhibited cell cycle processes, and increased apoptotic cells. The results of this study indicate that *C. sappan* is predicted to have activity as an anticancer agent.

Herbal medicines have several functions in pharmacology due to their bioactive compound content. This study presents that the *C. sappan* contains a high phenol and flavonoid content. The phenolic group, including flavonoids and tannins, has proven to be anticancer agents ^10^ and have antioxidant ^11^ activity. The results indicated that one of the bioactive compounds could have therapeutic activity for several diseases, e.g., antioxidant and anticancer activities.

*C. sappan* ethanol extract was proven to induce cell death in A549 cells. The cytotoxicity of the extract in A549 cells was 45.19 ± 1.704 μg/mL, indicating it has strong cytotoxicity. ^12^ This result is not far from the previous studies that treated T47D, PANC-1, and HeLa cells with *C. sappan* extract to obtain IC _50_ values of 68.00, 43.6, and 40.88 μg/mL, respectively. The extract could also significantly induce apoptosis by an analysis using Annexin-V. This apoptosis was verified by microscopic images and a high Bax/Bcl2 ratio. The apoptosis of A549 cells could be due to ATP depletion and mitochondrial dysfunction. Previous research has stated that hydroxy-chalcones group of compounds can cause ATP depletion, resulting in melanoma cell apoptosis. ^13^ *C. sappan* extract also caused A549 cell arrest in the G1 phase, which was also thought to be due to ATP depletion. ATP is required in the cell cycle for the phosphorylation of the cyclin/cyclin-dependent kinase complex. ^14^ The G1 phase is very sensitive to the availability of ATP. When the ATP content is too low, the cell will stop growing or prolong the duration of the phase until the amount of ATP is met. ^15^

ATP is the main energy source for cells because it has a phosphoanhydride bond that contains a lot of energy. Breaking this bond releases a huge amount of energy used for cellular processes. ^16^ ATP is required for DNA replication, protein biosynthesis, biochemical transport, protein activation, and pathways in cells. ^17^ ATP is synthesized in the mitochondria via oxidative phosphorylation that utilizes the proton gradient across the inner membrane of mitochondria. ^18^ The process involves electron transport, played by the protein complex I–IV. Downregulating proteins that play a role in electron transport can reduce ATP production, resulting in the disruption of various cellular processes. Previous research has suggested that prostate cancer cells can die due to ATP depletion *in vitro.* ^19^ In a previous study, downregulation of respiratory complex I mediated cell death. ^20^ Cell death that occurred in A549 cells due to the administration of the extract could be caused by ATP depletion and mitochondrial dysfunction.

The anticancer effect of *C. sappan* ethanol extract occurs via ATP depletion and mitochondrial dysfunction. The results of the functional annotation analysis of the RNA-seq showed that the proteins with decreased expression by sixfold compared to the control functioned as mitochondrial electron transport and ATP synthesis. Mitochondrial electron transport is played by the protein complex I–IV. RNA-seq results showed that in A549 cells, there was downregulation of the mitochondrial electron transport chain NADH to ubiquinone and the mitochondrial respiratory chain complex I assembly, both of which included protein complex I in the mitochondrial electron transport chain. Protein complex I transfers electrons from NADH to ubiquinone. Protein complex IV transfers electrons from cytochrome c to O _2_ to produce H _2_O. MET is played by protein complex I–IV. Meanwhile, upregulated genes have a role in cell adhesion and cell survival. Cell adhesion is needed by cancer cells for invasion and metastasis. Cellular adhesion is required for cell–cell interactions and interactions between cells with extracellular matrix components. ^21^ ATP depletion induces actin rearrangement, thereby reducing cell adhesion. ^22^ Cellular response to hypoxia is an adaptive response of cells due to a lack of oxygen to survive. ^23^ However, this biological process will not work in the presence of ATP depletion so that cells continue to undergo apoptosis, as happened in this study.

In addition to proteins that play a role in ATP synthesis, several proteins play a role in cancer growth that are downregulated. The downregulated protein is labeled in purple and in a larger font in Figure 4B. CD200R1 protein plays a role in modulating cancer immune microenvironments, which are generally overexpressed in NSCLC patients. ^24^ CD200R1, after binding to CD200, will suppress the antitumor response by modulating the activity of immune cells. ^25^ CEACAM7 is a protein that plays a role in cell adhesion. ^26^ RSPO4 is a protein that plays a role in activating the Wnt signaling pathway, which is an essential pathway for cell growth and development. ^27^ ARMCX1 plays a role in the regulation of mitochondrial transport. ^28^ The ZBTB8B protein interacts with proteins that play a role in mRNA processing and centrosome regulation during mitotic entry, namely, CPSF6, LSM, and KIF5B. ^29^^,^^30^ NXPH3 interacts with proteins related to the formation of microtubules such as TUBA1A and TUBA4A, where microtubules are indispensable during mitosis. ^31^ In addition, NXPH3 also interacts with CUL4A and the epithelial–mesenchymal transition-related protein that promotes cancer cell metastasis. ^32^ SOAT2 protein interacts with antiapoptotic protein BAG3. ^33^ FBXW12 interacts with SKP1 and IL22RA1, which plays a role in cell cycle progression and cancer cell growth induction. ^34^ Apart from downregulating genes, there are also several upregulating genes. As shown in the picture, upregulating genes play a role in survival, namely, CDH15, CLDN6, and FMOD, which have an important role in cell adhesion. ^35^^,^^36^ SCL8A3 and PDZD3 proteins play a role in maintaining intracellular ion homeostasis. ^37^ In addition, VARS2, which is upregulated, plays a role in mitochondrial protein synthesis. ^38^ However, the cell survival effort did not go well due to the ATP depletion caused by the administration of *C. sappan* extract.

## Conclusion

*C. sappan* has the capability to inhibit cancer cell growth by inducing apoptosis and downregulation of mitochondrial proteins on A549 cells, including ATP production, respiration, and mitochondrial function. The extract induced expression of genes that involved in the cell growth  and cell survival mechanism. However the A549 cells were going to apoptosis after treatment, since the mitochondrial protein that play important role in ATP production were shut down by the extract activity. Further research should be carried out to validate the physiological work of *C. sappan.*

## Data availability

### Underlying data

Zenodo: Anticancer effect of Caesalpinia sappan by downregulating mitochondrial genes on A549 lung cancer cell line. https://doi.org/10.5281/zenodo.5732978. ^39^

This project contains the following underlying data:

- C. sappan data.xlsx
- Enrichment RNA Seq C. sappan.xlsx

Data are available under the terms of the Creative Commons Attribution 4.0 International license (CC-BY 4.0).
