# Supplementary material for: Anticancer activity of Caesalpinia sappan by downregulating mitochondrial genes in A549 lung cancer cell line
Source: F1000Res. 2022 Aug 25;11:169. Originally published 2022 Feb 11. [Version 2] doi: 10.12688/f1000research.76187.2 (PMC9468624; doi:10.12688/f1000research.76187.2)
Supplement: Peer review history

# Peer review: Reviewer response for version 2

**DOI:** 10.5256/f1000research.137648.r148604

**Published:** 2022-09-12

**Authors:** Jayarani Fatimah Putri

**Affiliations:** 1 DAILAB, DBT-AIST International Center for Translational and Environmental Research (DAICENTER), National Institute of Advanced Industrial Science & Technology (AIST), Tsukuba, Japan

I have checked the manuscript and found that this article has answered and revised all the points I addressed.

Is the work clearly and accurately presented and does it cite the current literature?

Yes

If applicable, is the statistical analysis and its interpretation appropriate?

Partly

Are all the source data underlying the results available to ensure full reproducibility?

Yes

Is the study design appropriate and is the work technically sound?

Yes

Are the conclusions drawn adequately supported by the results?

Yes

Are sufficient details of methods and analysis provided to allow replication by others?

Yes

Reviewer Expertise:

My area of expertise is on Molecular Oncology (especially in molecular pathway and protein-protein interactions), Bioinformatics and Human Physiology.

I confirm that I have read this submission and believe that I have an appropriate level of expertise to confirm that it is of an acceptable scientific standard.

# Peer review: Reviewer response for version 1

**DOI:** 10.5256/f1000research.80153.r134490

**Published:** 2022-05-04

**Authors:** Jayarani Fatimah Putri

**Affiliations:** 1 DAILAB, DBT-AIST International Center for Translational and Environmental Research (DAICENTER), National Institute of Advanced Industrial Science & Technology (AIST), Tsukuba, Japan

General comments:

 This is an interesting study and the authors have used the cutting-edge methodology to address the answers. Generally, the paper is well written, clear, concise, and structured. The methods are generally appropriate with two possible exceptions. Clarification of a few details and provision of rationale of this particular method of transcriptomic sequencing should be provided. Also, the statistical analysis that was used should be addressed as well. Overall, the results are clear and compelling yet have a few details missing that are listed in specific comments below. The authors make a systematic contribution to the research literature in this area of investigation.

 Specific comments:

1. Repetitive sentences in the Introduction.
2. Cytotoxicity formula: the detail is missing.
3. The cell culture media and condition that was used is missing.
4. The second sentence on RNA extraction, preparation and transcriptome sequencing method does not match with the context.
5. The authors have established that IC50 (45,19ug/ml) value was used for apoptosis, cell cycle and Bax/Bcl2 assay. That would be make sense if IC50 value is covered for cell toxicity assay and graph. The dose range between 40-80ug/ml should be used to explain that.
6. The last sentence of fourth paragraph and first sentence of next paragraph after it is sounded vague. Removing any one sentence will be more reasonable.
7. The authors need to cite references in the discussion, especially in paragraph four.

Is the work clearly and accurately presented and does it cite the current literature?

Yes

If applicable, is the statistical analysis and its interpretation appropriate?

Partly

Are all the source data underlying the results available to ensure full reproducibility?

Yes

Is the study design appropriate and is the work technically sound?

Yes

Are the conclusions drawn adequately supported by the results?

Yes

Are sufficient details of methods and analysis provided to allow replication by others?

Yes

Reviewer Expertise:

My area of expertise is on Molecular Oncology (especially in molecular pathway and protein-protein interactions), Bioinformatics and Human Physiology.

I confirm that I have read this submission and believe that I have an appropriate level of expertise to confirm that it is of an acceptable scientific standard, however I have significant reservations, as outlined above.

# Peer review: Reviewer response for version 1

**DOI:** 10.5256/f1000research.80153.r124566

**Published:** 2022-03-09

**Authors:** Yi Li

**Affiliations:** 1 Johns Hopkins University, Baltimore, MD, USA

*C. sappan* to the A549 cancer cell line. Results indicate the *C. sappan* extract effectively slows cell growth and induces cell apoptosis. Gene expression analysis indicates the extract interferes with ATP synthesis in mitochondria, which leads to ATP depletion in cells. Overall, this study demonstrates the potential of using *C. sappan* as an anticancer agent by understanding *in vitro *cellular responses to different doses of *C. sappan*. The experiments included in this study are well designed and the methods are documented in excellent detail. The figures presented clearly present the results. This experiment evidence presented in this article strongly supports the conclusion, and the findings would provide important guidance to future works on understanding the molecular mechanisms of the biological effects of *C. sappan*.

 However, several previously published articles have investigated the effects of *C. sappan* to cancer cell lines ^1^ ^2^, including one on the same cell line (A549) ^3^.  Therefore, the major findings and conclusions presented in this article are not entirely novel. These previously published papers are not cited, and the previous findings were not discussed in the article. This article shows additional evidence that leads to findings included in previous studies, which is valuable to the researchers in the field but should not be branded as new findings as in the introduction section in this article. As the article is scientifically sound, I recommend the indexing of this paper, but I suggest the authors make changes in the main text to address this issue.

 Additional comments are below.

1. I assume the control group is using pure ethanol (0 ug/ml extract) in cell cultures but explicitly stating what control group condition is in the method section would be helpful.
2. “This result is not far from the previous studies that treated T47D, PANC-1, and HeLa cells with *C. sappan* extract to obtain IC50 values of 68.00, 43.6, and 40.88 μg/mL, respectively.” should have references.
3. Would including the antioxidant activity of some common antioxidant agents in Table 1 as comparisons help readers understand what the values entail?
4. Can authors comment on whether they expect the extract would also reduce ATP synthesis in healthy cells?

Is the work clearly and accurately presented and does it cite the current literature?

Partly

If applicable, is the statistical analysis and its interpretation appropriate?

Yes

Are all the source data underlying the results available to ensure full reproducibility?

Yes

Is the study design appropriate and is the work technically sound?

Yes

Are the conclusions drawn adequately supported by the results?

Yes

Are sufficient details of methods and analysis provided to allow replication by others?

Yes

Reviewer Expertise:

NA

I confirm that I have read this submission and believe that I have an appropriate level of expertise to confirm that it is of an acceptable scientific standard.

## References

[1] : Methanol extract from Vietnamese Caesalpinia sappan induces apoptosis in HeLa cells. *Biol Res* .2014;47: 10.1186/0717-6287-47-20 20 10.1186/0717-6287-47-20 25028057PMC4101718

[2] : Integrative Analysis to Uncover the Molecular Mechanisms ofCaesalpinia sappan L. for Anti-Cancer Activity. *Natural Product Communications* .2021;16(11) : 10.1177/1934578X211039922 10.1177/1934578X211039922

[3] : In vitro studies data on anticancer activity of Caesalpinia sappan L. heartwood and leaf extracts on MCF7 and A549 cell lines. *Data Brief* .2018;19: 10.1016/j.dib.2018.05.050 868-877 10.1016/j.dib.2018.05.050 29900385PMC5997910
